# Dual consistent pseudo label generation for multi-source domain adaptation without source data for medical image segmentation

**DOI:** 10.3389/fnins.2023.1209132

**Published:** 2023-06-26

**Authors:** Binke Cai, Liyan Ma, Yan Sun

**Affiliations:** School of Computer Engineering and Science, Shanghai University, Shanghai, China

**Keywords:** unsupervised domain adaptation, retinal vessel segmentation, semantic segmentation, multi-source, source-free

## Abstract

**Introduction:**

Unsupervised domain adaptation (UDA) aims to adapt a model learned from the source domain to the target domain. Thus, the model can obtain transferable knowledge even in target domain that does not have ground truth in this way. In medical image segmentation scenarios, there exist diverse data distributions caused by intensity in homogeneities and shape variabilities. But multi source data may not be freely accessible, especially medical images with patient identity information.

**Methods:**

To tackle this issue, we propose a new multi-source and source-free (MSSF) application scenario and a novel domain adaptation framework where in the training stage, we only get access to the well-trained source domain segmentation models without source data. First, we propose a new dual consistency constraint which uses domain-intra and domain-inter consistency to filter those predictions agreed by each individual domain expert and all domain experts. It can serve as a high-quality pseudo label generation method and produce correct supervised signals for target domain supervised learning. Next, we design a progressive entropy loss minimization method to minimize the class-inter distance of features, which is beneficial to enhance domain-intra and domain-inter consistency in turn.

**Results:**

Extensive experiments are performed for retinal vessel segmentation under MSSF condition and our approach produces impressive performance. The sensitivity metric of our approach is highest and it surpasses other methods with a large margin.

**Discussion:**

It is the first attempt to conduct researches on the retinal vessel segmentation task under multi-source and source-free scenarios. In medical applications, such adaptation method can avoid the privacy issue. Furthermore, how to balance the high sensitivity and high accuracy need to be further considered.

## 1. Introduction

Retinal diseases such as glaucoma and diabetic retinopathy often lead to blindness (Wu et al., 2021). It has been estimated that the risk of retinal-related diseases has increased greatly due to increasing pressure, lifestyle changes, and other potential factors. Such a trend pushes more and more researchers dedicated in exploring computer-aided diagnosis (CAD) systems for automatic and accurate diagnosis of retinal pathologies. It is of great significance for CAD systems to segment retinal vessels accurately because the segmentation result can provide the dependable diagnosis basis for examination of retinal diseases. Although there are a great quantity of classical model based methods about retinal vessel segmentation such as hand-crafted filters and fully connected conditional random fields (CRFs) (Orlando et al., 2016), it still remains challenging due to the large variation of the size of vessels, inhomogeneous lighting conditions, and other interference factors.

Semantic segmentation is one of the hot and widespread concerned topics in computer vision field, which aims to classify each pixel correctly in the whole image. Unprecedented advances in the semantic segmentation technique have been possible owning to the rapid development of convolutional neural networks (CNNs) and the availability of large-scale datasets. CNNs have outstanding ability to provide powerful and meaningful feature representations for medical image segmentation. Guo et al. (2023) proposed a new transformer framework based on CNN with parallax fusion paths for stereo image super-resolution. But there exists an obvious defect in the training process of supervised models that they entail a large training dataset equipped with labor-intensive annotations. The supervised models inevitably face challenges when they deal with new samples that correspond to different distributions with training samples. In medical image segmentation, the differences about the camera type and personal bioinformation lead to a distribution shift which hurts the performance of model in the target domain. Hence, how to transfer the knowledge of source model to the target domain is a significant problem for medical image analysis.

Recently, there has been extensive research about unsupervised domain adaptation (UDA) in the medical image segmentation field. On the one hand, some studies consider making maximum use of multiple source datasets to adapt a model from the source domain to the target domain (Kang et al., 2020; Li et al., 2021). Training with multiple source datasets can ease the condition of scarce expert knowledge ground truth. Furthermore, the adapted model is capable of exploring more essential knowledge with multiple source datasets involved. On the other hand, some studies propose to use the model's knowledge contained in the source model to transfer domain knowledge so as to preserve personal bioinformation in a medical image (Prabhu et al., 2021a; Yang et al., 2022). Medical data often cause problems about privacy as they contain sensitive information. Thus, source-free unsupervised domain adaptation (SFUDA) is a hot pot for medical applications where only the source trained model and target data are available.

Although those existing works have extremely promoted the possibility of real application for medical image segmentation, all of them only focus on one condition either non-source or multiple sources. Ahmed et al. (2021) explored such setting but they were devoted to the classification task. Therefore, we provide a more practical clinical setting where we have access to only the multiple source trained models in the adapting process for a segmentation task. In this multi-source and source-free setting (MSSF), it can not only protect patient's privacy but also make full use of multiple source datasets to learn more effective knowledge to eliminate distribution shift better. We measure the performance of our proposed method under multiple settings on three fundus image datasets. As far as we know, it is the first attempt to conduct researches on the retinal vessel segmentation task under multi-source and source-free scenarios.

## 2. Related work

Broadly, there are three different categories for unsupervised domain adaptations (UDAs) that include original unsupervised domain adaptation, source-free unsupervised domain adaptation (SFUDA), and multi-source unsupervised domain adaptation (MSUDA). Under the unsupervised domain adaptation scene, the goal of the model is to learn how to obtain more transferable features for the source domain and the target domain. It can be achieved by emphasizing the features of specific channels with less discrepancy between the first-order and second-order statistics of the source domain and target domain (Feng et al., 2021). Prabhu et al. (2021b) evaluated the reliability of a target instance based on its predictive consistency under a committee of random image transformations. Hoyer et al. (2022) proposed masked image consistency (MIC) that forces network to learn to infer the predictions of the masked regions from their context.

Medical data are sensitive, and they contain private bioinformation and identity information. It inevitably leads to privacy concerns during the process of adaptation with source data. Driven by this fact, some pseudo label generation methods use the knowledge of source model to denoise the pseudo label of target samples under source-free conditions (Chen et al., 2021; VS et al., 2022a). Bateson et al. (2022) introduced a label-free entropy loss and a domain-invariant prior that integrated in the form of a Kullback-Leibler divergence in loss function to guide the adaptation process. Yang et al. (2022) designed a Fourier Style Mining generator to inverse source-like images through statistic information. These generated images can simulate source data distribution and benefit the domain alignment. They designed a domain distillation loss to achieve feature-level adaptation and a domain contrastive loss to narrow down the domain shift using a self-supervised mechanism.

As depending on the characters of medical imaging instruments and patient' organs, medical image datasets from different sources follow different distributions. To make full use of the underlying values of multiple source datasets, adversarial learning is introduced to minimize the distribution shift between multiple source domains and target domains (Chen et al., 2021; VS et al., 2022a). He et al. (2021) proposed a simple image translation to align the pixel value distribution to reduce the domain shift. To make full use of unlabeled data, the pseudo labels generated by an ensembled model constrained the outputs of multiple source models. For the classification task, Ahmed et al. (2021) proposed a new domain adaptation strategy that the source models combine with suitable weights to predict a integrated classification result with the best quality than each source model.

## 3. Methods

### 3.1. Dataset description

**Figure 1:**
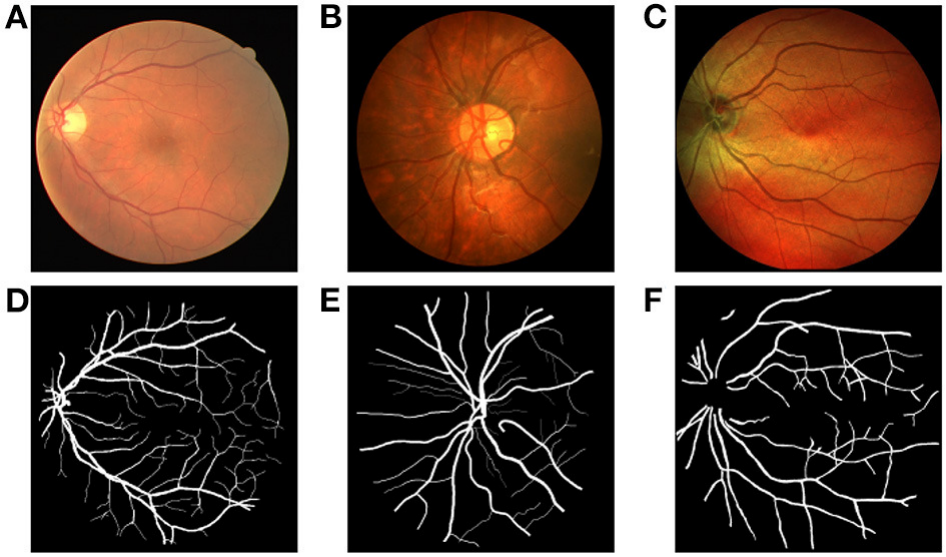
Example of fundus images and ground truths from the DRIVE, CHASEDB1, and IOSTAR datasets, respectively. **(A)** Fundus image 1 from DRIVE, **(B)** Fundus image 2 from CHASEDB1, **(C)** Fundus image 3 from IOSTAR, **(D)** ground truth of Fundus image 1, **(E)** ground truth of Fundus image 2, **(F)** ground truth of Fundus image 3.

In our experiments, we choose three public fundus image databases for evaluation including the DRIVE, CHASEDB1, and IOSTAR dataset (Figure 1). Each group of experiments chooses two databases as source domain data and the remaining one as target domain data. The DRIVE dataset contains 20 training images and 20 testing images. This dataset provides two labeled ground truths for each image, and we use the first labeled mask for training and testing. The CHASEDB1 (Child Heart and Health Study in England) dataset contains 28 color vascular images with a resolution of 990 × 960. There are two segmentation annotations available, and we adopt the first manual annotation in our study. We follow the setting in Li et al. (2015) and use the first 20 images for training and the remaining eight images for testing. The IOSTAR dataset includes 30 images taken with an EasyScan camera1 based on SLO technology. These high contrast images have a resolution of 1,024 × 1,024 with 45° FOV. The corresponding ground truths of these vessel images are annotated by experts having a good knowledge of retinal image analysis (Abbasi-Sureshjani et al., 2015; Zhang et al., 2016).

### 3.2. Measurement of performance

The retinal segmentation task is to classify each pixel in the fundus image into vessel pixel or background pixel. Obviously, it is a binary classification task. In order to analyze the performance of our proposed method quantitatively, we use several common metrics, including accuracy (Acc), sensitivity (Sen), specificity (Spe), which are defined as below:


(1) $$\begin{array}{l} A\text{cc}=\frac{TP+TN}{TP+FN+TN+FP}, \end{array}$$

(2) $$\begin{array}{l} Sen=\frac{TP}{TP+FN},Spe=\frac{TN}{TN+FP}, \end{array}$$

where TP and FP denote the number of foreground vessel pixels that are correctly segmented and the number of background pixels that are wrongly classified, respectively. TN represents the number of background pixels that are correctly segmented, and FN denotes the number of foreground vessel pixels that are wrongly classified as background class. Moreover, we also calculate the AUC metric (the area under the ROC curve) that is depended on the recall and precision and is more appropriate to measure performance under an unbalanced circumstance.

### 3.3. Approach

**Figure 2:**
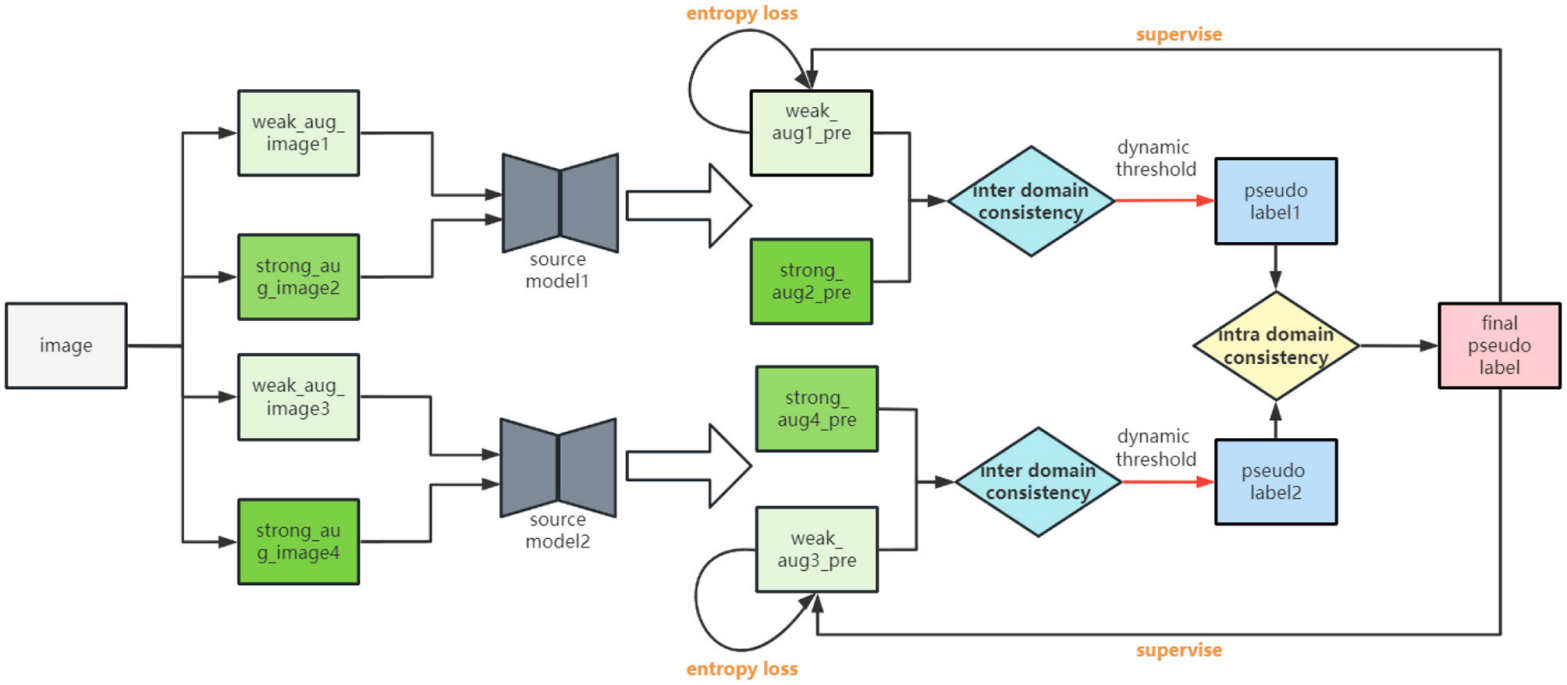
Overview of our proposed MSSF framework. Pseudo labels are generated under the guidance of the dual consistency constraint. Inter-domain consistency constraint aims to optimize the intra-class distance of each source model and also ease the distribution shift between the source domain and the target domain with two partially different predictions in a sense. Intra-domain consistency constraint can utilize the knowledge of multiple source models and thus can teach multiple models more essential and transferable knowledge regarding different data domains. Progressive entropy loss is complementary to our proposed dynamic pseudo label generation method, which can optimize the effectiveness of feature step by step.

Figure 2 illustrates the whole structure of our multi-source and source-free UDA framework. In this section, we first present the dual consistency mechanism including intra-domain consistency constraint and inter-domain consistency constraint. Next, we propose a progressive entropy loss that can optimize the features in a progressive way. The training procedures are finally presented.

#### 3.3.1. Inter-domain consistency constraint

Because of the presence of distribution shift, the model trained on the source domain tends to be frustrated when facing target sample. In order to deal with such problem, we introduce the inter-domain consistency constraint, which can select those reliable samples to improve the adaptation process.

For each target image, there are two different augmented images as the input of the source model, that is, a weak-augmented image and a strong-augmented image. The weak augmentation operations include intensity normalization, random rotation, and random flip. The strong augmentation operations include random gray scale adjustment and random color jitter besides the operations in weak augmentation. Therefore, we get two different prediction results for each pixel *i* in the two augmented images,


(3) $$\begin{array}{l} p_{i}^{\text{ks}}=S_{k}\left(x_{i}^{s}\right), \end{array}$$

(4) $$\begin{array}{l} p_{i}^{\text{kw}}=S_{k}\left(x_{i}^{w}\right), \end{array}$$

where *S*_*k*_ denotes the source model trained on the *k*th source domain. The superscript *s* denotes the strong augmentation and *w* denotes the weak augmentation. Due to the existence of domain shift between the source domain and the target domain in the early stage of model training, the two prediction results often have certain differences while the differences represent unreliable samples that do harm to the adaptation. Therefore, we introduce the inter-domain consistency constraint to discover credible samples. If the two prediction results at the same pixel position share the same category, this pixel sample is credible, and it can participate in the domain adaptation. On the contrary, this pixel sample is unreliable and should be discarded. Thus, we can obtain a consistency mask indicating the dependability of each pixel,


(5) $$m_{i}^{k}=\{\begin{array}{l} 1,l_{i}^{ks}==l_{i}^{kw},\\ 0,otherwise, \end{array}$$

where $l_{i}^{ks}$ and $l_{i}^{kw}$ denote the pseudo label of the *i*th pixel in strong-augmented image and weak-augmented image, and $m_{i}^{k}$ indicates whether the *i*th pixel is selected to adapt the *k*th source model.

For the approach of pseudo label generation, we proposed a dynamic threshold mechanism with weak-augmented image for each source model given by


(6) $$\begin{array}{l} T_{dyn}=\underset{\gamma \%}{\max}\left(sort\left(p_{i}^{kw}\right)\right). \end{array}$$

After ranking the predicted score results in ascending order, the top γ percentage probability value is taken as the dynamic factor. Dynamic factor fits in with the adaptation process that can adjust according to the training epoch,


(7) $$\begin{array}{l} \gamma =\min\left(a-\frac{b-a}{total\text{\_}epoch}*epoch,b\right), \end{array}$$

where a and b are the upper and lower bounds of the interval. With the increase of training rounds, the number of credible vessels samples in prediction also increases gradually, and the probability distribution of the prediction results gradually inclines to the high probability area. Thereafter, the dynamic threshold is supposed to be reduced. After the dynamic threshold is obtained, the final pseudo label can be obtained by


(8) $$l_{i}^{kw}=\{\begin{array}{l} \begin{array}{l} 1,p_{i}^{kw}>=T_{dyn},\\ 0,otherwise. \end{array} \end{array}$$

It should be noted that the generation method of pseudo label can be both applied to the prediction of weak-augmentation image and strong-augmentation image. The pseudo label of strong-augmentation image $l_{i}^{kw}$ only relates to the consistency mask $m_{i}^{k}$.

#### 3.3.2. Intra-domain consistency constraint

Considering that the inter-domain consistency constraint only focuses on a single source domain, it can only improve the feature compactness of the model in a single source domain. In order to make full use of the information in multiple source domains, we propose that the intra-domain consistency constraint learns the crucial knowledge and gets rid of the domain shift. Given two source domains and corresponding source models *S*_1_ and *S*_2_ as special cases, we can get the pseudo label imposing intra-domain consistency constraint for target sample,


(9) $$t_{i}=\{\begin{array}{l} l_{i}^{1w},l_{i}^{1w}==l_{i}^{2w},\\ 2,otherwise. \end{array}$$

The valid pseudo label *t*_*i*_ will be given to the pixel *i* only when the pseudo labels of *S*_1_ and *S*_2_ are consistent; otherwise, it will be assigned invalid category 2. The final output pseudo label only depends on the pseudo label of a weak-augmentation image instead of a strong-augmentation image.

Those highly reliable pixels obtained via inter-domain consistency constraint can maintain prediction-invariance on different source domain models for each target domain image. This prediction-invariance character can alleviate the domain shift between multiple source domains and target domains to a certain extent and improve generalization between multiple source domains and target domains. Therefore, for the dual consistency constraints, the intra-domain consistency constraint of a single source domain can reduce the intra-class distance and the feature space, while the intra-domain consistency constraint of multiple source domains can utilize multiple source domain models to ease the domain shift problem.

Thereafter, we introduce a consistency loss to utilize the advantages of both the inter-domain consistency constraint and intra-domain consistency constraint via filtering out samples that do not meet both consistency constraints. For all source domain models, consistency loss *Loss*_*con*_ is defined as


(10) $$\begin{array}{l} Loss_{con}=-\frac{1}{KN}\overset{K}{\underset{\text{k}=1}{\sum }}\overset{N}{\underset{i=1}{\sum }}CE\left(p_{i}^{kw},t_{i}\right), \end{array}$$

where CE denotes cross entropy loss,


(11) $$CE(p_{i}^{kw},t_{i})=\{\begin{array}{ll} -[p_{i}^{kw}logt_{i}+(1-p_{i}^{kw})log(1-t_{i})], & \;\\ t_{i}\neq 2\&m_{i}^{k}==1, & \;\\ 0,otherwise. & \; \end{array}$$

#### 3.3.3. Progressive entropy loss

Dual consistency constraints can identify valuable pixels for model training from both intra-domain and inter-domain perspectives to deal with the unlabeled data. However, this strategy is not completely satisfied. The pseudo label generated by dynamic threshold mechanism cannot entirely substitute for the real ground truth, which causes the intra-class feature to be not discriminative enough. Therefore, in order to further reduce the distance of the intra-class features, we propose progressive entropy loss.

Entropy minimization is familiar in semi-supervised learning and unsupervised domain adaptation, which essentially supervises model with the help of high probability regions during the training phase. On the other hand, entropy minimization can also be seen as a clustering method to compress the distance within each class, making the features extracted from the model more compact (Chen et al., 2019; Zou et al., 2019). However, there will be some problems occurring when applying this method directly. Due to the lack of ground truths, the model is usually unstable in the early training stage and the prediction is inaccurate. Then, the training model tends to collapse and fall into the local optimal solution. Therefore, we have come up with a progressive entropy loss strategy, which gradually increases the weight of the unsupervised entropy loss during the training phase to avoid the problem of insufficient optimization of the model.

First, the unsupervised entropy loss is calculated based on the prediction results of the weak augmented samples for multi-source models:


(12) $$\begin{array}{l} L{\text{oss}}_{ent}=-\frac{1}{KN}\overset{K}{\underset{k=1}{\sum }}\overset{N}{\underset{i=1}{\sum }}p_{i}^{kw}log\left(p_{i}^{kw}\right), \end{array}$$

where K denotes the number of source models and N denotes the whole pixel set of target dataset. The dynamic factor β will be adjusted according to the training epoch:


(13) $$\begin{array}{l} \beta =\max\left(a+\frac{b-a}{total\text{\_}epoch}*epoch,b\right). \end{array}$$

When the model gradually becomes stable with the increase of training epochs, it gradually strengthens the constraint of entropy minimization in a reasonable manner:


(14) $$\begin{array}{l} Loss_{pro\text{\_}ent}=\beta \times \left[-\frac{1}{KN}\overset{K}{\underset{k=1}{\sum }}\overset{N}{\underset{i=1}{\sum }}p_{i}^{kw}log\left(p_{i}^{kw}\right)\right]. \end{array}$$

Therefore, the final loss of our proposed method defined as follows:


(15) $$\begin{array}{l} Loss=Loss_{con}+Loss_{pro\text{\_}ent}. \end{array}$$

## 4. Experiments

### 4.1. Experiments setting

The implementation of our approach is based on the publicly Pytorch framework. We train our models on a NVIDIA GeForce RTX 3090 graphics card with a memory of 24 GB. We adopt the Adam algorithm as our network optimization method, of which the hyperparameters usually do not need to be adjusted.

Under the multi-source scenario, the number of source domain datasets in our experiments is 2, the batchsize is set to be 2 for both source domains, the training epoch is set to be 10, and the initial learning rate is set to be 0.00002. Because our experiments are conducted on the DRIVE, CHASEDB1, and IOSTAR datasets, in a multi-source scenario, three groups of experiments can be formed: (1) The source domains are the DRIVE and CHASEDB1 datasets, and the target domain is the IOSTAR dataset. (2) The source domains are the DRIVE and IOSTAR datasets, and the target domain is CHASEDB1. (3) The source domains are the CHASEDB1 and IOSTAR datasets, and the target domain is DRIVE. We evaluate all methods via four common metrics for segmentation task including AUC, accuracy (Acc), specificity (Spe), and sensitivity (Sen). The AUC represents the overall performance which is more appropriate to judge whether an algorithm is robust or not under an unbalanced circumstance. The higher the value of Acc, the higher the correct recognition rate of the algorithm. The Spe and Sen metrics indicate the recognition capacity of background class and vessel class, respectively.

### 4.2. Ablation experiments

We perform the ablation experiments to validate our proposed modules are effective or not on the DRIVE dataset. Comprehensive results are summarized in Table 1. The baseline method does not use any modules. It uses multiple source models to predict separately and then obtain pseudo labels for the prediction results of each source model directly through a hard threshold mechanism. The pseudo labels will be used to monitor the prediction results of target domain sample after the integration of the predicted results of multiple source domain models. This approach also utilizes knowledge from multiple source domains, similar to the idea of integrated learning. This method is also used as a strong baseline under multi-source scenarios in our comparison study. The method-a adds inter-domain consistency constraint module (inter-domain CC) based on the baseline. The method-b adds the intra-domain CC constraint module (Intra-domain CC) based on method-a. The method-c adds progressive entropy loss (PEL) based on method-b.

**Table 1:** Ablation study on DRIVE dataset.

**Method**	**Inter-domain CC**	**Intra-domain CC**	**PEL**	**AUC**	**Acc**	**Spe**	**Sen**
Baseline	–	–	–	0.9737	**0.9660**	**0.9830**	0.7793
Method-a	✓	–	–	0.9755	0.9652	0.9819	0.7916
Method-b	✓	✓	–	0.9762	0.9623	0.9741	0.8384
Method-c	✓	✓	✓	**0.9764**	0.9611	0.9718	**0.8493**

The bold values indicate the highest performance metrics in each column.

#### 4.2.1. The impact of inter-domain consistency constraint

Inter-domain consistency constraint can filter out pixels with inconsistent categories in the predicted results under different augmentation operations, improving the stability and consistency of the model. Such constraint can explore more valuable vessel samples than background samples. Therefore, it has a strengthening effect on the learning of vessel regions, and the sensitivity of the method-a is improved compared to the baseline.

#### 4.2.2. The impact of intra-domain consistency constraint

When there is only the inter-domain consistency constraint module, the knowledge of each source domain model is mixed, which is not beneficial to the learning of knowledge in the target domain. Accordingly, when introducing the intra-domain consistency constraint module, more effective vessel pixels are identified during the model training for supervised learning, making full use of the inherent knowledge of multiple source domains. Therefore, the AUC and sensitivity metrics of method-b are increased compared with method-a, especially the increase in sensitivity. However, the specificity decreases from 0.9819 to 0.9741 due to such constraint, because it filters some samples of background class when the model is able to identify more vessels.

#### 4.2.3. The impact of progressive entropy loss

On the one hand, unsupervised progressive entropy loss enhances the compactness of intra-class features. The high probability regions obtained through supervised learning with pseudo labels guide the model to extract more discriminative features for background and vessel classes. On the other hand, because the generation of pseudo label is based on the dynamic threshold mechanism, it gradually strengthens the recognition capability of vessels during the adaptation process. Therefore, compared with the other experiment group, method-c has a significant improvement in sensitivity, with the highest AUC and sensitivity. Although the accuracy and specificity of the final model have slightly decreased, it has brought about significant improvements in sensitivity, which is more practical for medical image segmentation and can detect more foreground objects to assist in medical diagnosis.

**Figure 3:**
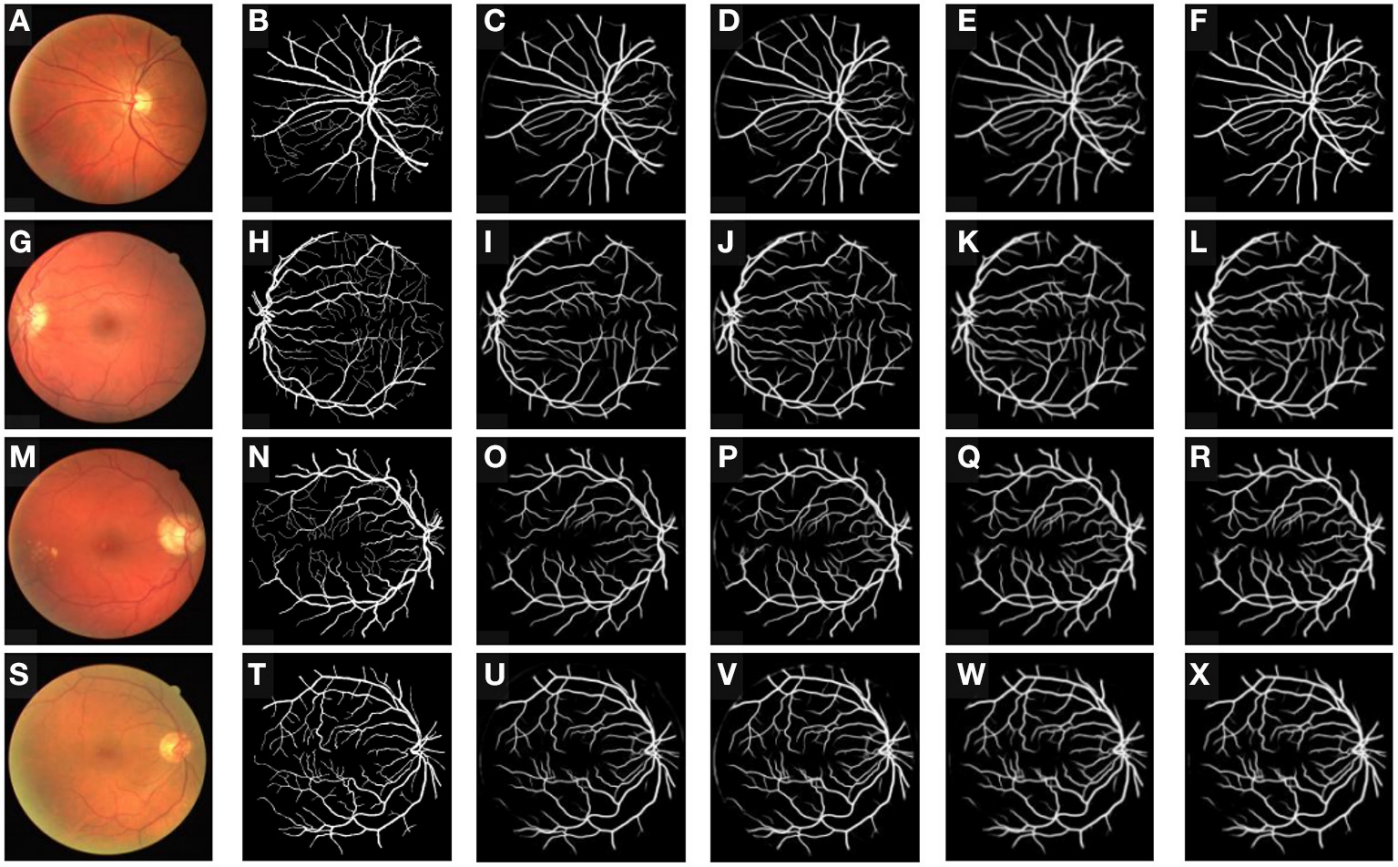
Retinal vessel segmentation results for our proposed method on the DRIVE dataset. From the column in left to right **(A–X)**, the retinal original images, the ground truths, the segmentation results of the baseline, the segmentation results of method-a, and the segmentation results of method-b, the segmentation results of method-c.

We also provide the visualization result of our proposed method in different ablation experiment groups in Figure 3. It can be seen that for method-a group with only the inter-domain consistency constraint module, it is easy to predict the outer circle of the eyeball as a blood vessel, indicating that the pseudo labels for blood vessels are not accurate enough, and the features extracted from the model are not clean. The introducing of the intra-domain consistency constraint module greatly improves this problem because it can filter out pixels that are prone to false segmentation by using the knowledge of multiple source models. Since progressive entropy loss can be beneficial to obtain more discriminative features, it can be found that the method-c recognizes more difficult samples correctly.

### 4.3. Comparison experiments

We perform experiments on DRIVE, CHASEDB1 and IOSTAR three datasets, where we choose two datasets as the source domain and the remaining one as the target domain. We compare our proposed method with three methods with three different multi-source and source-free single condition settings in Table 2.

**Table 2:** Comparison experiments on three target domains.

**Method**	**AUC**	**Acc**	**Spe**	**Sen**
**Source domains: drive and CHASEDB1, target Domain:IOSTAR**				
Oracle	0.9850	0.9658	0.9831	0.8426
AdaptSegNet (DRIVE/CHASEDB1)	0.9361/0.9680	0.9534/0.9569	0.9802/**0.9812**	0.6943/0.6703
DPL (DRIVE/CHASEDB1)	0.9569/0.9631	0.9458/0.9499	0.9771/0.9783	0.7027/0.7124
TT_SFUDA (DRIVE/CHASEDB1)	0.9104/0.9344	0.9390/0.9358	0.9799/0.9774	0.6218/0.6496
Multi-Source	**0.9834**	**0.9651**	0.9789	0.8180
Ours	0.9824	0.9625	0.9743	**0.8357**
**Source domains: drive and IOSTAR, target Domain:CHASEDB1**				
Oracle	0.9883	0.9711	0.9776	0.8769
AdaptSegNet (DRIVE/IOSTAR)	0.9659/0.9388	0.9561/0.9533	0.9853/0.9839	0.7824/0.6930
DPL (DRIVE/IOSTAR)	0.9513/0.9652	0.9511/**0.9630**	0.9842/**0.9861**	0.6397/0.7442
TT_SFUDA (DRIVE/IOSTAR)	0.9517/0.9556	0.9396/0.9393	0.9714/0.9710	0.7793/0.7871
Multi-Source	**0.9819**	0.9616	0.9688	0.8546
Ours	0.9816	0.9606	0.9674	**0.8601**
**Source domain: CHASEDB1 and IOSTAR, target Domain:DRIVE**				
Oracle	0.9833	0.9631	0.9738	0.8516
AdaptSegNet (CHASEDB1/IOSTAR)	0.9638/0.9470	0.9591/0.9512	**0.9877**/0.9843	0.6634/0.6789
DPL (CHASEDB1/IOSTAR)	0.9511/0.9553	0.9501/0.9528	0.9816/0.9845	0.6332/0.6351
TT_SFUDA (CHASEDB1/IOSTAR)	0.9314/0.9407	0.9336/0.9389	0.9759/0.9801	0.7768/0.7598
Multi-Source	0.9737	**0.9660**	0.9830	0.7793
Ours	**0.9764**	0.9611	0.9718	**0.8493**

The bold values indicate the highest performance metrics in each column.

Compared to the original unsupervised domain adaptation method such as AdaptSegNet (Tsai et al., 2018), adversarial learning at the output result level is clearly desirable due to the similar spatial location and target sizes in cityscape dataset. However, there are significant differences and complex distribution in different vessels, and it failed to capture the effective knowledge of vessel distribution. On the other hand, our proposed method achieves better performance than two source-free domain adaptation methods including DPL and TT_SFUDA (Chen et al., 2019; VS et al., 2022b). These two methods do not essentially solve the domain shift problem because of the significant differences in experimental results across the different target domains. Due to the existence of multiple source models, our proposed method can alleviate the domain shift on the target domain through dual consistency constraints and sufficiently explore the essential knowledge of multiple source domains. Therefore, it is minimally affected by the magnitude of the domain shift, and has gained relatively ideal performance in different target domains. The performance of the multi-source algorithm is familiar with our method, but when the target domain is DRIVE with a large number of thin vessels, its performance drops a lot. Such a defect can be attributed to the lack of effective treatment of the pseudo label.

Our proposed method achieves better performance than unsupervised domain adaptation methods including source-free and multi-source single scene settings on different target domains. It can sufficiently explore the knowledge fusion in multiple source models while retaining the advantage of source pretrained model of high AUC and sensitivity metrics. Such advantage makes our approach more meaningful and practical that more vessels can be identified as correctly as possible especially under the unsupervised domain adaptation scenario.

**Figure 4:**
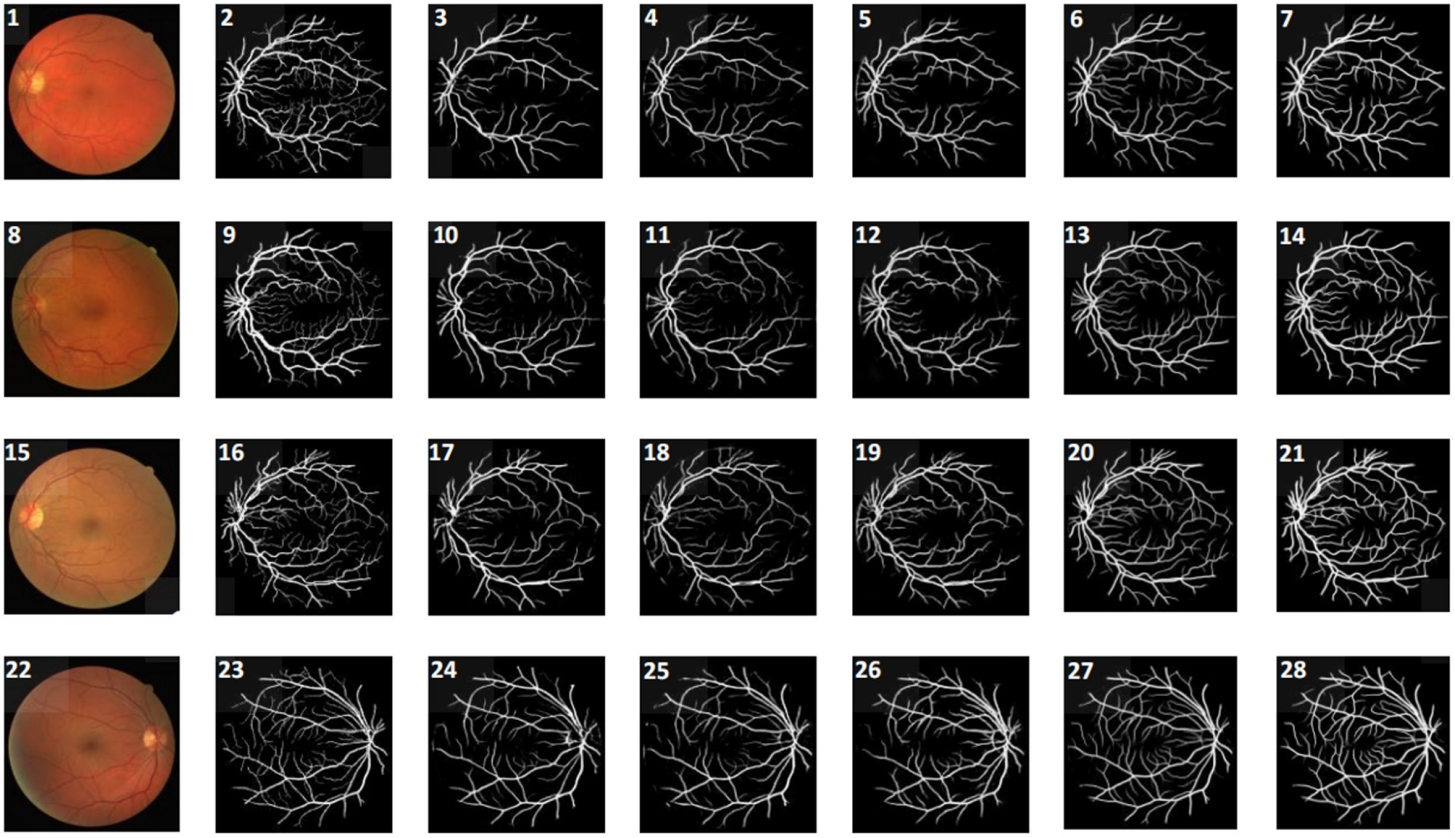
Visualization for different methods on DRIVE target domain. From the **left** to **right** columns, they are original image, ground truth, and the segmentation results of AdaptSegNet, DPL, TT_SFUDA, multi-source, and our proposed MSSF algorithm, respectively.

We also present a visual comparison of the segmentation results of several methods as shown in Figure 4. Compared with other methods, our approach has fewer false segmentation cases, which effectively avoids the occurrence of mistakenly identifying the outer circle of the eye as vessel class. It also has the best recognition performance for a large number of capillaries in the middle area of a fundus image, preventing the fracture of vessel occurring.

## 5. Conclusion

This study designs a brand-new unsupervised domain adaptation framework, which expands the single unsupervised domain adaptation scene including source-free and multi-source settings. Our proposed dual consistency constraint can filter out noisy pseudo labels based on the knowledge in each source models and the fusion between them. To effectively promote the feature clustering, progressive entropy loss can not only compress the distance within each class but also can benefit the generation of pseudo label in turn. The proposed MSSF framework combines the advantages of source-free and multi-source adaptation. We hope this paradigm can inspire future studies about unsupervised domain adaptation.

## Data availability statement

Publicly available datasets were analyzed in this study. This data can be found at: https://drive.grand-challenge.org/, http://www.retinacheck.org/download-iostar-retinalvessel-segmentation-dataset, and https://blogs.kingston.ac.uk/retinal/chasedb1/.

## Ethics statement

Written informed consent was obtained from the individual(s), and minor(s)' legal guardian/next of kin, for the publication of any potentially identifiable images or data included in this article.

## Author contributions

BC: conceptualization, methodology, software, investigation, formal analysis, and writing-original draft. LM: data curation, methodology, resources, software, supervision, writing-original draft. YS: visualization and investigation. All authors contributed to the article and approved the submitted version.
